# Propagating the missing bacteriophages: a large bacteriophage in a new class

**DOI:** 10.1186/1743-422X-4-21

**Published:** 2007-02-26

**Authors:** Philip Serwer, Shirley J Hayes, Julie A Thomas, Stephen C Hardies

**Affiliations:** 1 Department of Biochemistry, The University of Texas Health Science Center at San Antonio, 7703 Floyd Curl Drive, San Antonio, Texas, 78229-3900, USA

## Abstract

The number of successful propagations/isolations of soil-borne bacteriophages is small in comparison to the number of bacteriophages observed by microscopy (great plaque count anomaly). As one resolution of the great plaque count anomaly, we use propagation in ultra-dilute agarose gels to isolate a *Bacillus thuringiensis *bacteriophage with a large head (95 nm in diameter), tail (486 × 26 nm), corkscrew-like tail fibers (187 × 10 nm) and genome (221 Kb) that cannot be detected by the usual procedures of microbiology. This new bacteriophage, called 0305φ8-36 (first number is month/year of isolation; remaining two numbers identify the host and bacteriophage), has a high dependence of plaque size on the concentration of a supporting agarose gel. Bacteriophage 0305φ8-36 does not propagate in the traditional gels used for bacteriophage plaque formation and also does not produce visible lysis of liquid cultures. Bacteriophage 0305φ8-36 aggregates and, during *de novo *isolation from the environment, is likely to be invisible to procedures of physical detection that use either filtration or centrifugal pelleting to remove bacteria. Bacteriophage 0305φ8-36 is in a new genomic class, based on genes for both structural components and DNA packaging ATPase. Thus, knowledge of environmental virus diversity is expanded with prospect of greater future expansion.

## Findings

Current data indicate that roughly 10^31 ^bacteriophages exist worldwide, including about 10^8 ^genotypes and possibly most of the earth's gene diversity [1-4]. These estimates are derived from either fluorescence or electron microscopy. Less than 1% of the observed bacteriophages have ever been grown in culture (sometimes called "the great plaque count anomaly" [1-4]). The great plaque count anomaly is especially dramatic in the case of soil-borne bacteriophages. Propagated bacteriophages are sometimes not obtained from soil samples in spite of concentrations in the 10^8 ^– 10^9 ^range per gram, when detected by microscopy [5]. As shown below, some bacteriophages, though viable, are probably not detected by any past procedures. Genomes of currently unpropagated bacteriophages are potentially a major source of unexplored environmental gene diversity.

Knowledge of environmental virus gene diversity recently has been most expanded by sequencing of large eukaryotic phycodnaviruses and related viruses. These viruses have double-stranded DNA genomes with a length between 200 and 1,200 Kb [6-9]. Large double-stranded DNA bacteriophages also exist, including *Bacillus megaterium *bacteriophage G (~670 Kb genome [10]), *Pseudomonas aeruginosa *bacteriophage φKZ (280 Kb genome [11]) and several bacteriophages that are relatives of bacteriophage T4 by the criteria of DNA replication/recombination strategy, structure and interface of DNA replication to DNA packaging [12,13].

However, of the 5,400 or so bacteriophages that have been isolated [14] (96% have double-stranded DNA genomes) and of 405 deposited in databases [15], only 6 (4 T4-like) have genomes as long as 200 Kb. Two other T4-like bacteriophage genomes in draft status are also in this range [12]. Statistical analysis reveals a significant under-sampling of long-genome bacteriophages [6]. The strong possibility exists that long-genome bacteriophages (>200 Kb genome) are more frequent and are major contributors to microbial ecology, but are under-sampled because of the use of classical bacteriophage propagation procedures and possibly also classical processing of environmental samples for microscopy. For example, bacteriophage G was discovered by accident ~40 years ago through electron microscopy of a preparation of another bacteriophage [16]. Thus, we raise the question of whether a major pool of environmental bacteriophages remains undetected.

**Figure 1:**
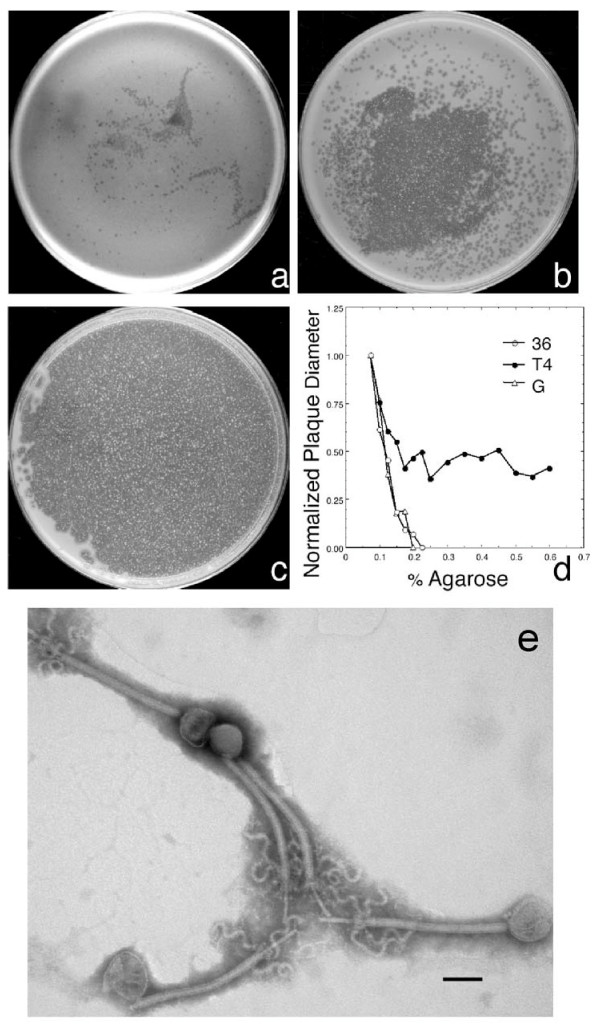
Screening and electron microscopy of bacteriophage 0305φ8-36. Bacteriophage 0305φ8-36 was initially propagated and isolated [17] from soil frequented by cattle at the King Ranch (Kingsville, Texas). The host was a locally isolated *Bacillus *that was typed as *B. thuringiensis *by sequencing of the gene for 16s ribosomal DNA, as previously described [17]. During isolation, single-plaque cloning was performed [17] in gels of 0.40%, 0.20% and 0.15% agarose. The inocula for all three Petri plates were bacteriophages from a single plaque of the previous propagation, transferred by sterile needle and then non-uniformly spread [17]. The three Petri plates were at the same temperature (±0.2 C) during incubation. Photographic images are shown of Petri plates used for propagation in agarose gels of the following percentages: (a) 0.4, (b) 0.20, (c) 0.15. (d) In a more comprehensive experiment, plots of plaque diameter as a function of agarose gel percentage were made for bacteriophages G, T4 and 0305φ8-36 (0305φ8-36 is abbreviated by 36 in the figure). The molten agarose solution was the same among the different bacteriophages in (d). The host for bacteriophage G was *Bacillus megaterium*; the host for T4 was *Escherichia coli *BB/1. All Petri plates for (d) were in contact with the same surface and the temperature did not vary among them by more than 0.2°C. (e) Electron microscopy was performed of bacteriophage 0305φ8-36 negatively stained with sodium phosphotungstate after purification from a plate stock by use of a cesium chloride step gradient [17]. The length of the bar is 0.1 μm; magnification calibration was checked with a diffraction grating. The tails of all bacteriophage particles have partially contracted. By this criterion, 0305φ8-36 is a myovirus.

To probe the pool of comparatively large environmental bacteriophages, in the present study, extraction and propagation were performed in comparatively dilute, 0.15% agarose gels. The gels contained 10 g Bacto tryptone, 5 g KCl in 1000 ml water with 0.002 M CaCl_2 _added post-autoclaving [17]. Numerous bacteriophages were screened during single plaque cloning by determining the change in plaque size with change in supporting agarose gel concentration. *Bacillus thuringiensis *bacteriophage 0305φ8-36 made small (<1 mm) plaques in a 0.4% agarose supporting gel (Figure 1a). Plaques became progressively larger as the agarose gel concentration decreased to 0.2% (Figure 1b) and 0.15% (Figure 1c; plaques are seen at the left; most of the plate is confluent). This dependence is comparatively steep, as confirmed in a side-by-side comparison with bacteriophages T4 and G (Figure 1d). Post-isolation, 0305φ8-36 grew only in gels of either 0.25% or more dilute agarose. Thus, 0305φ8-36 was assumed to be comparatively large and was selected for further study.

Bacteriophage 0305φ8-36 was, indeed, comparatively large. Electron microscopy of a negatively stained specimen of purified bacteriophage particles (Figure 1e) revealed a contractile-tail virus (myovirus)[18,19] with a polyhedral DNA-containing capsid that had a diameter of 95 ± 4 nm. In addition, bacteriophage 0305φ8-36 had (a) a tail that was long, 486 ± 23 nm in length and 26 ± 3 nm in diameter, in comparison to those for other *Myoviridae *[20], and (b) tail fibers that were also comparatively large, 187 ± 13 nm in length and 10 ± 1 nm in diameter. Bacteriophage tail fiber diameter has been generally conserved at about 2 nm among other tailed bacteriophages [20]. In addition, the tail fibers had an unusual sine wave-like appearance in projection and are presumably corkscrew-like in three dimensions. The genome of 0305φ8-36 was correspondingly large (221 Kb) by pulsed field gel electrophoresis (PFGE) (not shown). Reports of bacteriophages with morphology of this general type have previously appeared [21]. But, to the authors' knowledge, further investigation was not performed.

The purified bacteriophage 0305φ8-36 particles in Figure 1e are in contact with each other, although most of the specimen is empty (not shown). This feature was reproducible and is explained by aggregation. This level of aggregation is not characteristic of either bacteriophage T4 or bacteriophage G (see ref. [22] for G). Analytical velocity centrifugation (B. Demeler, J. Thomas, S.C. Hardies and P. Serwer, unpublished observations) confirms aggregation via a sedimentation coefficient that varies continuously between 350 and 1,200. Fluorescence microscopy of material removed from plaques reveals that aggregation also occurs during growth (not shown).

Whatever the details of aggregation, aggregates were potential contributors to the steep dependence of plaque size on supporting agarose gel concentration (Figure 1d). Aggregates must, however, dissociate during dilution because plaque forming efficiency per DNA molecule was over 0.5 when the concentration of DNA molecules was determined from ethidium-stained DNA fluorescence after expulsion of DNA molecules from capsids and PFGE. Possibly, aggregation assists stabilization in harsh conditions. Before its extraction and isolation, bacteriophage 0305φ8-36 had been dry in the laboratory for 7 months.

The unusual biology of 0305φ8-36 is accompanied by an unusual genome, based on sequence determination. For example, the 0305φ8-36 DNA packaging ATPase was identified by use of the SAM HMM procedures previously described [17] with E = 5.17e-54. Motifs found and aligned include the following: (1) ATPase motif, including adenine-binding motif, P-loop motif, and DExx box [23] and (2) conserved aspartate residues of the endonuclease ruvC fold [24]. The aligned 0305φ8-36 DNA packaging ATPase intersects the homology tree for this protein [17] only at the center. That is to say, no other known DNA packaging ATPase is in the same class. Most other genes are too diverged from known genes to identify. A few 0305φ8-36 genes for myovirus structural components have been identified, but without any indication of membership in any previously known group (data not shown). Comprehensive analysis of the 0305φ8-36 genomic sequence is in progress.

Without the dilute gel propagation used here, bacteriophage 0305φ8-36 and its accompanying novelty would probably have been inaccessible to detection because the classical detection procedures, i.e., community sequencing [25], liquid enrichment culture and microscopy [26], are not expected to work for the following reasons:

(a) In addition to not growing in the 0.4 – 0.7% agarose gels classically used [26] for plaque formation, bacteriophage 0305φ8-36 does not produce visible lysis of liquid cultures. Thus, liquid enrichment cultures [26] would be ineffective at detection. Titers of 2–3 × 10^9 ^plaque-forming units per ml were achieved at 25°C during growth in an aerated liquid culture. The culture had been inoculated at a multiplicity of 0.01, based on observed bacteriophage titer. The bacteriophage growth proceeded with a lag of 100 min. and then a rapid growth phase of ~260 min. (apparent burst size = 22–30 after 60 min.), followed by a period of slower growth that ended at ~1,440 min. (24 hr.). Bacteria overgrew the culture without any visible lysis and these bacteria were 0305φ8-36-resistant (5 independent bacterial clones). The cause for growth limitation in liquid culture is not known, but a likely cause is aggregation that lowers the infection rate when the bacteriophage reaches 2–3 × 10^9 ^per ml.

(b) Community sequencing, fluorescence microscopy and electron microscopy are performed on preparations from which μm-sized particles like bacteria are usually removed by either centrifugation or filtration ([4]; reviewed in ref. [26]). These procedures will also remove aggregates like those of bacteriophage 0305φ8-36 and thus are also expected to be ineffective.

The data presented here show that (a) some bacteriophages in the uncultivatable category can now be moved to the cultivatable category and (b) a new category must be added for aggregating viruses not yet detected by any procedure. Given the heterogeneity of the geology and bacterial microbiology of soil particles even within a single sample [27,28], multiple niches can be envisaged for independent bacteriophage evolution even in a single sample. Thus, the various soil niches have the potential to produce genomic diversity significantly above current estimates. Access to at least some of this diversity is now expanded.

## Competing interests

The author(s) declare that they have no competing interests.

## Authors' contributions

PS isolated bacteriophages, designed the study and wrote the manuscript. SJH performed the plaque size vs. gel percentage experiments and the PFGE. JT performed the sequencing, informatics analysis and some of the background research. SCH supervised the sequencing and informatic analysis.
